# Supplementary material for: Clinical and Pathological Risk Factors for Peritoneal Metastases in a Surgical Series of T4 Colorectal Cancers
Source: Cancers (Basel). 2025 Mar 25;17(7):1103. doi: 10.3390/cancers17071103 (PMC11988146; doi:10.3390/cancers17071103)
Supplement: Supplementary file 1 [file cancers-17-01103-s001.zip › cancers-3469384-supplementary.pdf]

Supplementary Table S1. Pathological and biological baseline characteristics according to the presence vs. absence of synchronous peritoneal metastases.

Variables	Categories	N.	%	Synchronous peritoneal metastases				
				yes (n = 73)		no (n = 279)		*p*
				N.	%	N.	%	value
Histological type	Intestinal	279	80.2	47	16.8	232	83.2	<0.001
	Mucinous/other	69	19.8	26	37.3	43	62.3	
Histological grading	Well diff.	3	0.9	1	33.3	2	66.7	0.095
	Moderately diff.	129	38.4	21	16.3	108	83.7	
	Poorly diff.	184	54.7	46	25.0	138	75.0	
Sampled nodes	≤ 12	58	16.7	13	20.7	46	79.3	1.000
	>12	290	83.3	57	20.0	232	80.0	
Ulceration	Present	236	90.4	43	89.6	193	90.6	0.789
	Absent	25	9.6	5	10.4	20	9.4	
Intratumoral vasc. invasion	Present	230	83.0	42	18.2	188	81.8	0.839
	Absent	47	27.0	9	19.2	38	80.8	
Extratumoral vasc. invasion	Present	223	80.5	44	23.2	179	76.8	1.000
	Absent	54	19.5	11	21.4	43	79.6	
Neural invasion	Present	219	79.1	43	19.6	176	80.4	0.851
	Absent	58	20.9	10	17.2	48	82.8	
Pattern of invasiveness	Infiltrative	220	81.5	44	20.0	176	80.0	0.424
	Expansive	50	18.5	7	14.0	43	86.0	
Peritumoral infiltrat. lymphocytes	Absent/Mild	130	48.1	35	26.9	95	73.1	0.001
	Moderate/Severe	140	51.9	15	10.7	125	89.3	
Intratumoral infiltrat. lymphocytes	Absent/mild	167	62.3	40	24.0	127	76.0	0.002
	Moderate/severe	101	27.7	9	8.9	92	91.1	
Crohn's like lymphoid reaction	Present	38	14.7	4	10.5	34	89.5	0.183
	Absent	221	85.3	45	20.4	176	79.6	
Resection margins	R0	294	86.5	52	17.7	242	82.3	0.571
	R1	32	9.4	9	18.1	23	71.9	
	R2	14	4.1	3	21.4	11	78.6	
Microsatellite status	MSS/pMMR	146	84.4	45	30.8	101	69.2	0.466
	MSI-H/dMMR	23	13.6	5	21.7	18	78.3	
KRAS	Mutated	75	42.6	27	36.0	48	64.0	0.752
	Wild type	101	57.4	34	33.7	67	66.3	
NRAS	Mutated	6	4.1	0	-	6	100.0	0.082
	Wild type	141	95.9	57	40.4	84	59.6	
BRAF	Mutated	16	9.5	5	31.3	11	68.7	0.791
	Wild type	151	90.5	54	35.7	97	64.3	

NA: not available/not assessed; IQ: inter quartile; SRC: signet ring cell; R0: microscopically free; R1: microscopically involved; R2: macroscopically involved;; MSS: microsatellite stability; MSI-H: microsatellite instability high; pMMR: mismatch repair proficiency; dMMR: mismatch repair deficiency.
